# Bioactive Compounds from Fruit and Vegetable Waste: Extraction and Possible Utilization

**DOI:** 10.3390/foods13050775

**Published:** 2024-03-01

**Authors:** Noelia Castillejo, Lorena Martínez-Zamora

**Affiliations:** 1 Department of Agricultural Sciences, Food, Natural Resources and Engineering, University of Foggia, Via Napoli 25, I-71122 Foggia, Italy; 2 Department of Food Technology, Nutrition and Food Science, Faculty of Veterinary Sciences, University of Murcia, Espinardo, 30071 Murcia, Spain; 3 Postharvest and Refrigeration Group, Department of Agronomical Engineering and Institute of Plant Biotechnology, Universidad Politécnica de Cartagena, Cartagena, 30203 Murcia, Spain

## 1. Introduction

Globally, there is a serious problem with fruit and vegetable waste, which can result from improper food handling or storage techniques or from the disposal of inedible portions of produce. Approximately one-third of worldwide production is lost or wasted at some point in the food chain, according to reports from the Food and Agriculture Organization (FAO) [1]. One of the Sustainable Development Goals (SDGs), reducing food waste by 50%, is one of the FAO’s major challenges for 2050. Waste is considered the raw material for a circular economy, which is based on the idea of a “zero waste” economy driven by society [2].

By-products from fruits and vegetables can be employed as innovative ingredients or food fortifiers because they are high in bioactive components [3]. Just as we may cut down on the quantity of wasted fruit and vegetable matter, an ideal extraction of these health-promoting components will enable efficient utilization of these compounds [4]. Additionally, these by-products support a circular economy because they have a wide range of possible uses in various industries [5]. By doing so, we can enhance food security and nutrition while creating more sustainable farming systems.

Therefore, the purpose of this Special Issue is to showcase current developments in cutting-edge green technology for the extraction of bioactive substances from fruit and vegetable by-products, along with their possible applications. To achieve this purpose, leading researchers from all over the world contributed to this Special Issue by providing new methods to address this problem.

## 2. An Overview of the Published Articles

Thirteen scientific works are included in this Special Issue, with them focusing on the optimization of the extraction of bioactive compounds, the characterization of several by-products, the development of new food products, and the evaluation of the functionality of these extracts.

Reis et al. [contribution 1] optimized the extraction of *Passiflora cincinnata*, which belongs to the same genus as passion fruit, to obtain several bioproducts such as oil rich in linoleic acid, antioxidant extracts, and oil microparticles with antioxidant extracts. The seeds were pressed, and the best extraction condition (solid–liquid ratio 1:48 with 58% ethanol at 74 °C) produced an extract rich in lignans, with high antioxidant capacity and antimicrobial activity against Gram-positive and Gram-negative bacteria. In addition, the oil was microencapsulated and enriched with *Passiflora cincinnata* antioxidant extracts, improving the oxidation stability of the product by up to 30%.

In this line of research, Sai-Ut et al. [contribution 2] optimized the extraction of phenolic compounds from lychee and longan seeds using response surface methodology (RSM). The best condition for maximizing extraction yield, phenolic content, and antioxidant activity using a 1:20 solid–liquid ratio was 41% and 53% ethanol at 51 °C and 58 °C for 139 min and 220 min for lychee and longan seeds, respectively.

Moreover, Afifi et al. [contribution 3] characterized the main metabolites present in orange peels (albedo and flavedo) and juices from different countries. Sixty-six metabolites were detected through the use of nano-liquid chromatography coupled to a high-resolution electrospray-ionization quadrupole time-of-flight mass spectrometer. In *Citrus sinensis*, eleven metabolites were discovered for the first time. Certain flavonoids were significantly more abundant in the citrus peel than in the juice, suggesting that the peel had greater potential for its use in industry and the circular economy. The huge number of metabolites found in the peel, which is the main by-product during fruit processing, means that it should be considered for its use as a new ingredient in the food industry.

Within the development of new food products, Salas-Millán et al. [contribution 4] developed a melon-based sparkling wine with 12% *v*/*v* ethanol. Within the volatile profile, the compounds isoamyl acetate, ethyl decanoate, 3,6-nonadienyl acetate, and (E,Z)-nonadien-1-ol contributed to the sweet, fruity, banana, tropical, nutty, and melon aroma. The sensory evaluation of this melon-based wine at the end of its shelf-life scored 92 points, with 100 being the maximum. This study shows how by-product revalorization can yield new products with good sensory acceptance, market potential, and a distinctive aroma, such as the aforementioned innovative sparkling wine.

Following this topic, Pinho et al. [contribution 5] focused their research on the extraction of carotenoids from guarana peels, and their encapsulation via spray drying to improve their stability. The 1:2 ratio (concentrated ethanolic extract:gum arabic) was added to oatmeal paste. The minimum temperature applied was 70 °C, with the temperature reaching up to 90 °C. The oatmeal paste enriched with carotenoid microparticles showed lower viscosity because the presence of the microparticles reduced the accessible water. However, pigment loss decreased with encapsulation. These findings imply that the production of stable and useful products may be possible with the addition of encapsulated carotenoids to cooked foods at higher temperatures.

Also, in this line of research, Pérez et al. [contribution 6] studied the revalorization of broccoli and carrot by-products, formulating a new vegetable drink. Although three types of preservative treatments were applied, namely pasteurization, ultrasound, and high hydrostatic pressure, only the last treatment process was able to show the best results, maintaining a high concentration of carotenoids and sulfur compounds, which contributed to prolonging its shelf-life, increasing its antioxidant capacity, and reducing its microbial load, respectively. 

Besides the determination and quantification of the main bioactive compounds in the by-products, it is crucial to determine the effect of these compounds on health. Hence, Olennikov et al. [contribution 7] characterized the waste generated by the lingonberry processing industry and its potential functionality. The lipid profile and antioxidant status of hamsters fed with a high-fat diet ended up being normalized by polysaccharides from lingonberry, according to the results of in vitro and in vivo experiments. These results provide credence to the idea that food processing waste can be used to produce hypolipidemic and antioxidant substances in the pharmaceutical sector.

Additionally, in this research line, Han et al. [contribution 8] studied the hepatoprotective effects of tamarind shell extract using zebrafish and chicken embryos as in vitro models. The use of tamarind shell extracts made it possible to successfully reverse the ethanol-induced pathological alterations, liver dysfunction, and ethanol–metabolic enzyme malfunction in the chick embryo liver, zebrafish, and HepG2 cells. Tamarind extract suppressed the level of excess reactive oxygen species in zebrafish and HepG2 cells and restored the altered potential of the mitochondrial membrane. This suggested that tamarind shell extract attenuates alcohol liver disease by activating NRF2 to suppress ethanol-induced oxidative stress.

Furthermore, Coelho et al. [contribution 9] studied a potential prebiotic ingredient consisting of tomato flour (seed and peel) rich in phenolic compounds and carotenoids extracted via ohmic and conventional extraction. For this purpose, the flour was subjected to simulated gastrointestinal digestion, and its fermentability potential and impact on gut microbiota were determined. The probiotic strain SFCONV favored the growth of *Bifidobacterium animalis*, while SFOH favored the growth of *Bifidobacterium longum*, probably due to the different carbohydrate profiles of the flours. Overall, the flours used were able to function as a direct substrate to favor the potential prebiotic growth of *Bifidobacterium longum*. The results of the fecal fermentation model showed the highest growth of Bacteroidetes with SFOH and the highest values of Bacteroides with SFCONV.

In this line of research, Zhou et al. [contribution 10] also studied the functionality of walnut kernel extracts (defatted or whole), measuring antioxidant activity in vitro and in vivo. The defatted walnut kernel extract showed the highest antioxidant activity under extraction conditions with 58% ethanol at 48 °C for 77 min.

In addition, similar to this topic, Phumat et al. [contribution 11] studied the functionality of *Benincasa hispida* peel extracts from consumer residues, with the extracts obtained from 95% ethanol showing the best results in terms of anti-aging and antioxidant activity. They highlighted the absence of toxicity and the irritant effect of this extract, which is promising for the development of new products.

In addition, two scientific reviews are included in this collection. The characterization of the main bioactive compounds found in several by-products is important to understand how they can be re-utilized. In this sense, as editors of this Special Issue, Martínez-Zamora et al. [contribution 12] presented a systematic review of lemon by-products as new flavonoid-rich ingredients, analyzing their extraction using green technologies and the incorporation of these flavonoid-rich extracts into new foods. This analysis reported that 89% of the papers studied used green technologies and solvents for extraction and 18 papers were related to the reuse of these extracts, although only 35% of these works evaluated the functionality of such incorporation.

Lastly, Chetrariu and Dabija [contribution 13] presented an updated review on spent grain and its use as an ingredient in various food products. This food is high in fats, fiber, protein, vitamins, and minerals. Because of its high moisture content and microbial sensitivity, it needs to be processed using an appropriate, economical, and ecologically friendly recovery approach. This by-product, due to its nutritional properties, is utilized as a raw material to make a variety of food products, including frankfurters, fruit drinks, pasta, pastries, muffins, waffles, yogurt and plant-based yogurt substitutes, and other snacks. The circular economy finds opportunities in the regeneration and recycling of waste materials and energy that become inputs to other food processes.

Therefore, the extraction and potential utilization of bioactive compounds from fruit and vegetable waste have emerged as a promising and relevant research field in agro-food waste management [6]. This area provides a unique opportunity to address both food loss reduction and the implementation of more sustainable and environmentally friendly practices [7]. Bioactive compounds derived from these waste materials can contribute to human nutrition and health and facilitate the creation of value-added products, thus promoting circular economy principles [8].

## 3. Conclusions and Future Perspectives

In summary, exploring bioactive compounds from fruit and vegetable waste presents a promising avenue in agro-food waste management, offering opportunities to reduce food loss and adopt sustainable practices. These compounds, with potential benefits for human nutrition and health, open doors for the development of value-added products. The ongoing exploration and development of new extraction techniques and innovative applications by young researchers are crucial to maximize the potential of this research avenue, with a view to a prosperous future in the areas of enhanced sustainability and comprehensive waste management in the food industry.

## Data Availability

Not applicable.
